# Supplementary material for: Temporal control of late replication and coordination of origin firing by self-stabilizing Rif1-PP1 hubs in Drosophila
Source: Proc Natl Acad Sci U S A. 2022 Jun 22;119(26):e2200780119. doi: 10.1073/pnas.2200780119 (PMC9245680; doi:10.1073/pnas.2200780119)
Supplement: Supplementary File [file pnas.2200780119.sapp.pdf]

Supplementary Information:

Temporal control of late replication and coordination of origin firing by self-stabilizing Rif1-PP1 hubs in *Drosophila*

Chun-Yi Cho,[1] Charles A. Seller,[1] and Patrick H. O'Farrell [1,*]

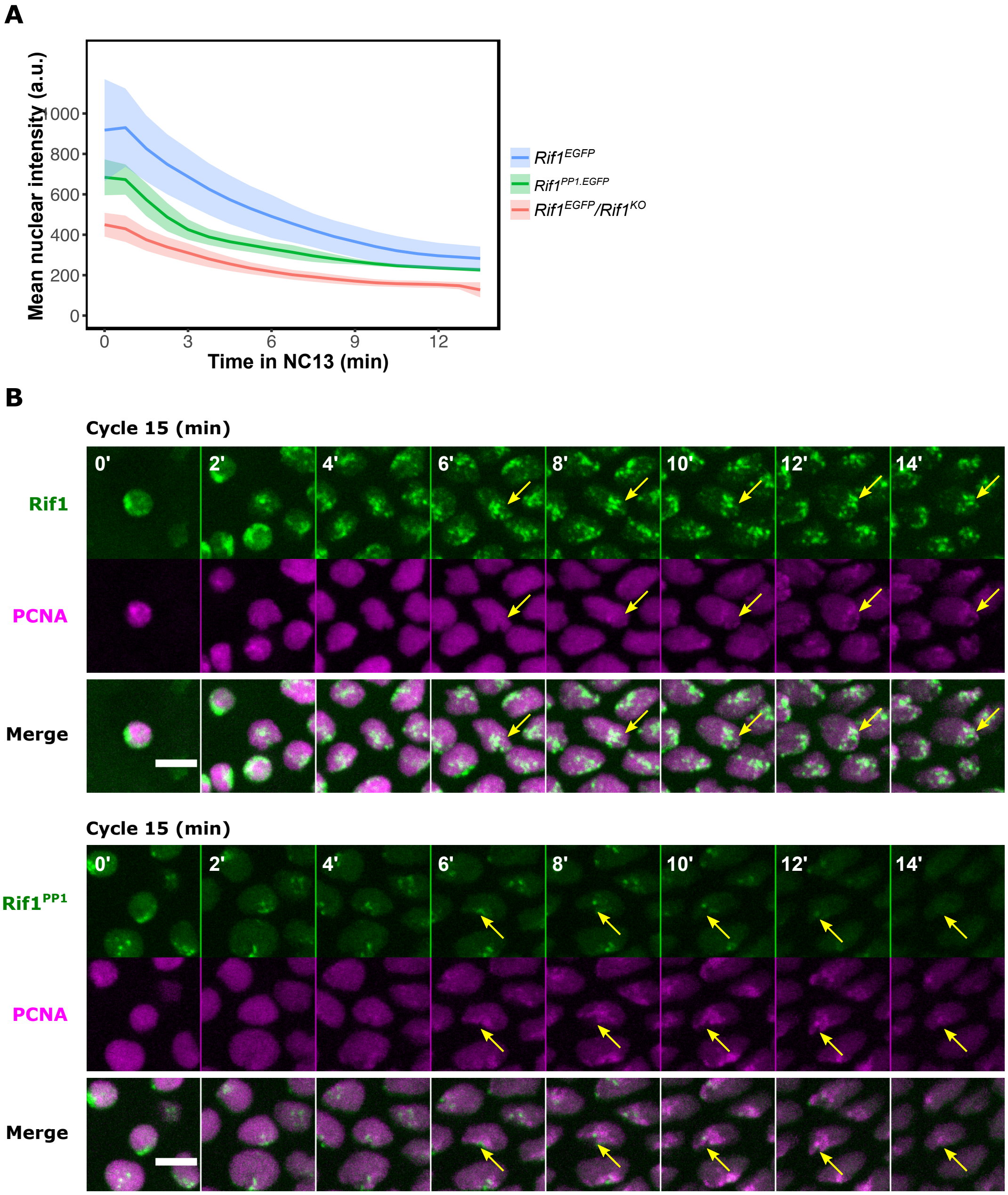

Figure S1. Additional analyses related to Figure 1. (A) Mean fluorescent intensity of GFP-tagged Rif1 or Rif1^{PP1} in indicated genotype during NC13. Data are mean ± SD (n = 5 embryos). a.u., arbitrary unit. (B) Time-lapse imaging of GFP-tagged Rif1 or Rif1^{pp1} along with mCherry-PCNA during cycle 15. Arrows point to the chromocenter at the apical region of the nucleus. Note the early loss of Rif1^{PP1} from the chromocenter compared to the persistence of Rif1. These maximal projection images lack the resolution to distinguish the distinct localization of PCNA and Rif1 signals in this area of the nuclei described previously. The start of interphase is referred to as time point 0'. Scale bars, 6 μm.

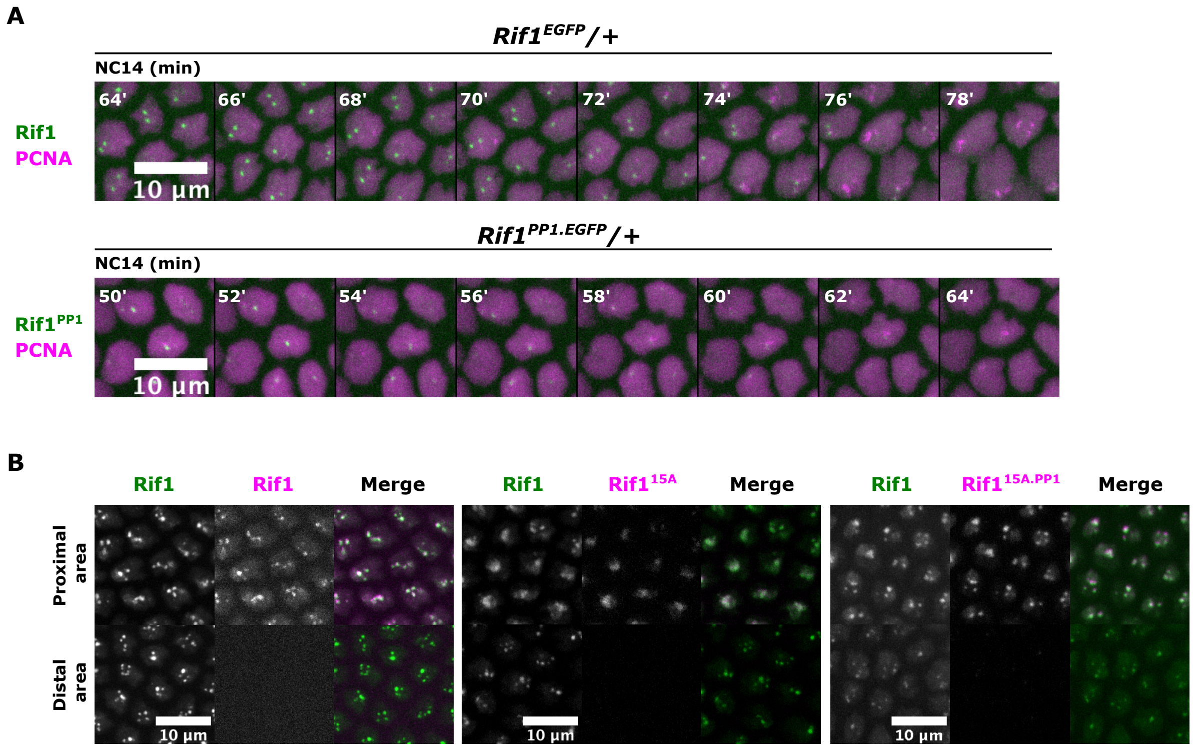

Figure S2. Additional analyses related to Figure 2. (A) Time-lapse imaging of embryos from females of indicated genotypes during late NC14 when Rif1-GFP foci were dissociating. The start of interphase 14 is set as time point 0'. (B) Snapshots from time-lapse movies following Rif1-GFP embryos injected with mRNAs encoding various alleles of Rif1 tagged with mScarlet-I. Nuclei proximal or distal to injection sites about 1 hour in S phase 14 are shown. Similar results were observed in at least 5 embryos for each experiment.

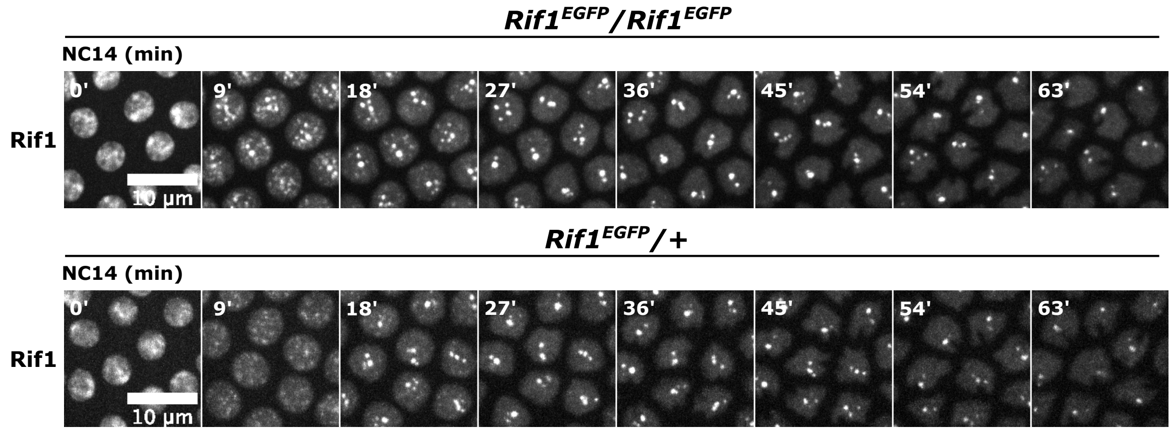

Figure S3. Additional analyses related to Figure 3. Time-lapse imaging of Rif1-GFP in embryos from females carrying one or two copies of *Rif1*^{EGFP} allele during NC14.

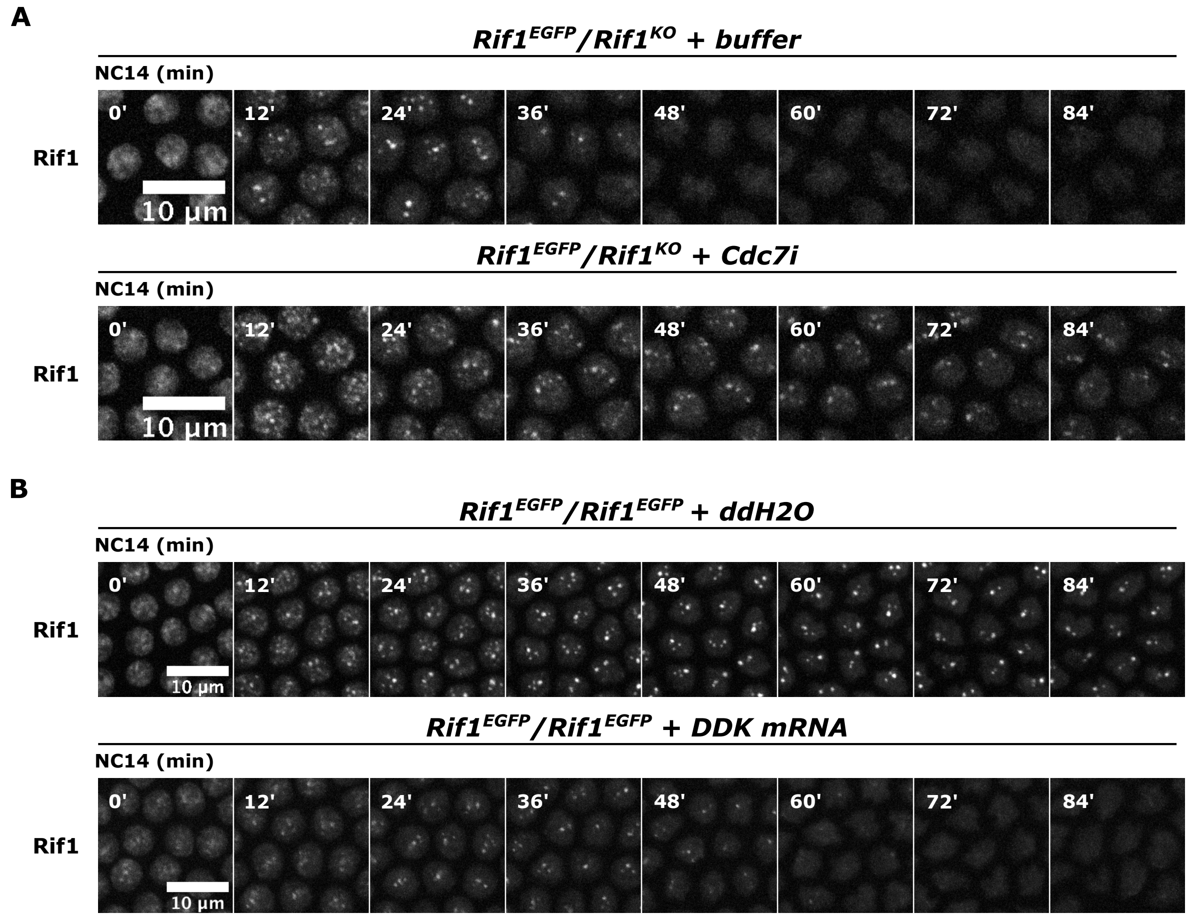

Figure S4. Additional analyses related to Figure 4. (A) Time-lapse imaging of Rif1-GFP in cycle 14 embryos from *Rif1*^{EGFP}/*Rif1*^{KO} heterozygous females injected with either buffer or Cdc7i. The injection of Cdc7i was performed during mitosis 13. (B) Time-lapse imaging of Rif1-GFP in cycle 14 embryos from *Rif1*^{EGFP} homozygous females injected with either buffer or mRNAs encoding DDK subunits. The injections were performed before cycle 13.

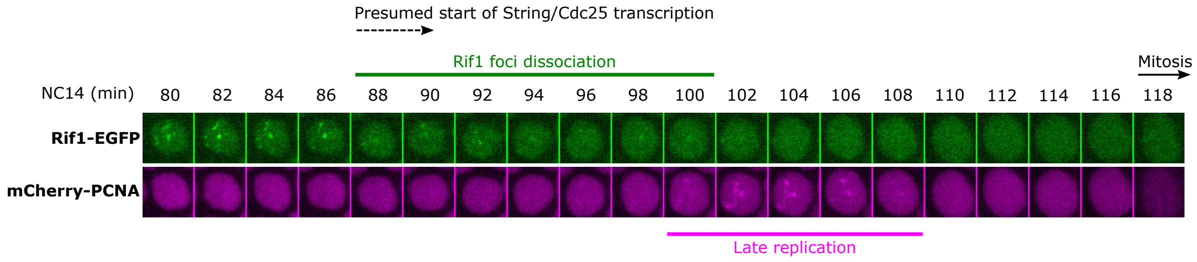

Figure S5. Partial inhibition of Cdc7 results in a second wave of replication controlled by a “fail-safe” program associated with the mitotic entry program. Snapshots captured from Movie S1 focusing on a nucleus in the embryo injected with Cdc7i during late NC14. The time of String/Cdc25 expression is taken from measurements of the lag between the first detectable zygotic transcription and onset of mitosis (37).

Movie S1. Inhibition of Cdc7 kinase activity stabilizes Rif1 foci during cycle 14. Time-lapse imaging of Rif1-GFP and mCherry-PCNA in a cycle 14 embryo collected from heterozygous $Rif1^{EGFP}/Rif1^{KO}$ females and injected with either buffer or Cdc7i. Areas near the cephalic furrow are shown with anterior pole toward the left.
